# Context Sensitive Modeling of Cancer Drug Sensitivity

**DOI:** 10.1371/journal.pone.0133850

**Published:** 2015-08-14

**Authors:** Bo-Juen Chen, Oren Litvin, Lyle Ungar, Dana Pe’er

**Affiliations:** 1 Department of Biomedical Informatics, Columbia University, New York, New York, 10032, United States of America; 2 Department of Biological Sciences, Department of Systems Biology, Columbia University, New York, New York, 10027, United States of America; 3 Computer and Information Science, University of Pennsylvania, Philadelphia, Pennsylvania, 19104, United States of America; University of Erlangen-Nuremberg, GERMANY

## Abstract

Recent screening of drug sensitivity in large panels of cancer cell lines provides a valuable resource towards developing algorithms that predict drug response. Since more samples provide increased statistical power, most approaches to prediction of drug sensitivity pool multiple cancer types together without distinction. However, pan-cancer results can be misleading due to the confounding effects of tissues or cancer subtypes. On the other hand, independent analysis for each cancer-type is hampered by small sample size. To balance this trade-off, we present CHER (Contextual Heterogeneity Enabled Regression), an algorithm that builds predictive models for drug sensitivity by selecting predictive genomic features and deciding which ones should—and should not—be shared across different cancers, tissues and drugs. CHER provides significantly more accurate models of drug sensitivity than comparable elastic-net-based models. Moreover, CHER provides better insight into the underlying biological processes by finding a sparse set of shared and type-specific genomic features.

## Introduction

With the recent advances in next-generation sequencing technologies, the prospects of personalized healthcare look brighter than ever [1]. The use of genomics to guide clinical care is perhaps most widespread in cancer [2, 3]. Many pioneer studies have shown how one can use signatures of gene expression to predict clinical outcomes for individual patients [4–6]. More recently two large collections of matched drug screens and genomics profiles of cancer cell lines have been published [7, 8]. These data have been used to build predictive models of drug response by associating genomic features with drug sensitivity in cancer cell lines [9–12]. Additionally, connecting drug sensitivity to specific genomic features can help shed light on the mechanisms of drug action and elucidate the underlying reasons for resistance to the treatment. Thus, these data offer the opportunity to develop methods that can be used for personalized treatment.

A key challenge in associating genetic characteristics to drug sensitivity is the role of context in biological systems. For example, regulation of gene expression has been shown to have patterns specific to tissues and cell-types [13–16]. In tumorigenesis, diverse patterns of mutation, gene expression, and epigenetic regulation have also been observed in cancer-specific or tissue-specific manner [17, 18]. This context dependency plays an important role in the efficacy of treatment. For example, PLX4732, a RAF inhibitor targeting oncogenic *BRAF* ^*V600E*^, is a potent treatment for melanoma patients with the mutation [19]. However, colon cancer patients with the same mutation do not respond to PLX4732 [20]. It is therefore important to take into account the context created by cancer types when analyzing the genomics of drug sensitivity.

It is no surprise that predictive models built using only melanoma data give better prediction for melanoma samples than those built using data of mixed cancer types [7]. This argues that we should focus on one cancer type when building models for drug sensitivity. While such strategy allows us to avoid confounding influence of context, it constrains us to a small number of samples. Due to sample size, current datasets lack the statistical power to build separate models for each cancer.

We utilize commonality between cancer types and drugs to overcome the paucity of data. We propose CHER (Contextual Heterogeneity Enabled Regression), an algorithm that builds predictive models by selecting genomic features and deciding which ones are shared or not between cancer types, tissues and drugs. CHER is empowered by two assumptions. First, CHER assumes similar cancer types may have similar mechanisms underlying drug sensitivity. For example, basal-like breast cancer and ovarian cancer share many molecular signatures [21]; therefore, these two cancers are likely to share similar predictive genomic features for drug sensitivity. Second, CHER assumes that if two drugs induce similar responses, their predictive models are likely similar. These assumptions allow CHER to boost its power to uncover biomarkers predictive of drug sensitivity by sharing information between cancers and drugs.

We applied CHER to three datasets from the Cancer Cell Line Encyclopedia (CCLE) [7] and demonstrate that CHER gives significantly more accurate modeling of drug sensitivity in these datasets compared to other methods. Contrary to previous methods that assume all samples have the same predictive features, CHER explicitly learns which predictive features should be shared or not between cancers or subtypes. For data with multiple subtypes of samples, CHER also identifies the relevant subtype that dictates the context specificity, offering the potential to shed light onto mechanisms underlying pharmacogenomics.

Below we first present the motivation and concept of CHER, followed by the results from the application to CCLE data. We then compare CHER’s performance with other methods and demonstrate CHER’s superior performance. Example models from CHER are showcased and discussed. Details about CHER algorithm are then presented in Materials and Methods and S1 Text.

## Results

### Contextual Heterogeneity Enabled Regression

We use data from Cancer Cell Line Encyclopedia (CCLE) [7] for our analysis. The CCLE cohort includes 36 different cancer types that are typically pooled together for analysis with no distinction between types [7]. However, effects of tissue on drug sensitivity are evident (S1 Fig).

One way to tackle this issue is to regress out the mean effect of tissues through multivariate analysis of variance (MANOVA) and then model the residuals of all samples together [8]. However, this does not take care of the contextual effect. That is, the effect of tissue-gene interactions. For example, *MDM2* overexpression is known to be predictive of sensitivity to Nutlin-3 in acute myeloid leukemia [22] and acute lymphoblastic leukemia [23]. However, the correlation between *MDM2* expression and sensitivity to Nutlin-3 varies greatly between tissues (Pearson’s correlation coefficient r: -0.01 ~ -0.53). S2B Fig shows the association between *MDM2* expression and sensitivity to Nutlin-3 in different tissues. Although this association can be detected using all samples (r = -0.38, p < 5e-8), such association is misleading, as *MDM2* expression does not have any predictive power for tissues as such lung or pancreas (S2B Fig). Moreover, if we discard samples from those tissues where the association is absent, we can see enhanced association (S2A Fig) and an increase in *MDM2’s* predictive power in these tissues. As each tissue might have different degrees of association between *MDM2* expression and sensitivity to Nutlin-3, such tissue-specific gene effects will become *tissue-gene interaction effects* when all samples are pooled together for analysis. Using MANOVA to simply regress out the average effect of each tissue will not resolve such tissue-specific gene effect.

Ideally we would limit the analysis to one cancer type at a time, but unfortunately the resulting sample size is currently too small. The available drug sensitivity data in CLLE includes fewer than 40 samples for most cancers, except lung cancer (n = 91), cancers originated from haematopoietic and lymphoid tissues (n = 70), and skin cancer (n = 40) (S3 Fig) and even these sample sizes are relatively small. The lack of statistical power due to small sample size is further exacerbated by the size and complexity of the human genome.

To gain statistical power and still account for context specificity we developed *CHER* (Contextual Heterogeneity Enabled Regression), an algorithm based on transfer learning [24] that selects predictive genomic features and builds regression models for drug sensitivity. Unlike other algorithms, CHER aims to uncover predictive features that are shared across contexts, as well as features that are predictive only in certain contexts. A context can be a cancer type, tissue type, or cancer subtype. We refer to this context as the *relevant subtype*, or the *split*, that separates individuals into two groups where the predictive program of drug sensitivity can be different.

CHER simultaneously achieves two goals: CHER explicitly performs sparse feature selection while optimizing performance of prediction of drug sensitivity. Whereas optimizing prediction of drug sensitivity prediction is crucial for precision medicine, sparse feature selection allows for biological interpretation of the resulting models. The latter is especially important because it may provide an understanding of drug resistance that could shed light on ways to improve drug development or combinatorial therapy.

**Fig 1:**
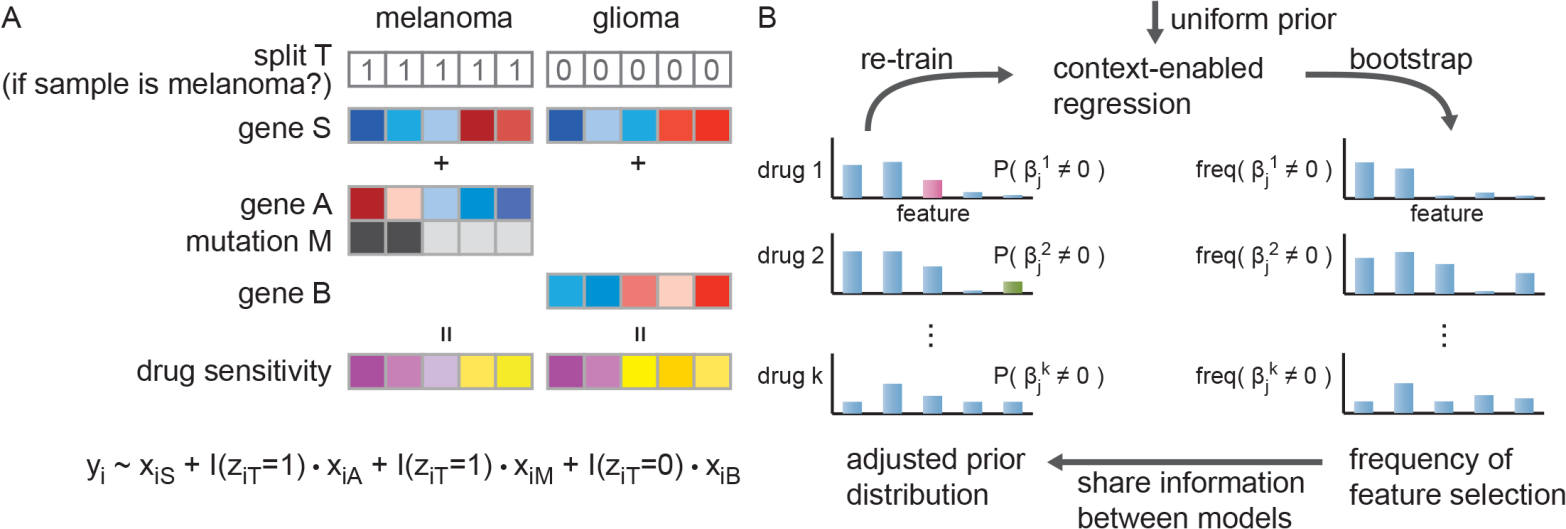
Overview of CHER algorithm. A. Example of a model learned by CHER, where the drug sensitivity of melanoma samples can be predicted by mutation of M and gene expression of A and S, whereas in glioma, expression of gene S and B are the predictors. CHER takes advantage of pooling samples together to gain statistical power, identifying both shared (gene S) and context-specific features (A, B and M). In cases where the relevant context is unknown, the algorithm searches for the best “split”, if any, to separate samples into two groups. Yi represents drug sensitivity of the ith sample, xi are the corresponding features of the ith sample, zit = 1 presents the ith sample is melanoma, and I(.) is an indicator function. B. Iterative learning scheme of CHER. CHER initially learns models with uniform prior (meaning each genomic feature has the same probability of being included in the model). During each iteration, CHER trains the regression models with bootstrapping, which allows the algorithm to establish the frequency of each feature being selected. Then CHER adjusts the priors according to the distribution of frequency and the similarity between phenotypes.

Our algorithm is inspired by transfer learning theory [24]. We increase power by sharing information between cancers and between drugs. First, we learn models from similar cancers, essentially sharing the information between cancers by assuming that they may share the same genomic features responsible for drug sensitivity (Fig 1A). By pooling samples of similar cancers, we boost power to learn predictors common to them. To learn context-specific, or cancer-type specific predictors, we introduce a *split* variable that represents types/subtypes of cancers. This split variable conditions the predictive effects of context-specific features via interaction terms between the split variable and the predictors in the model (for examples, gene A and mutation M in melanoma, Fig 1A). Note, the choice of split is part of the optimization problem. CHER learns how to separate samples into two groups, when such separation of samples increases predictive power. At this stage, CHER has learned an initial model that may contain both predictors that are shared between cancers or specific to one of them.

Next, we boost CHER’s learning by transferring information between drugs (Fig 1B). We assume that if two drugs induce a similar response, their predictive models are likely similar as well. For example, if two drugs induce highly correlated responses and we have observed gene *A* as a predictor for sensitivity to one drug, it is more likely gene *A* is also predictive for the other drug. This allows us to adjust our belief of each feature being predictive of drug sensitivity by comparing models derived for similar drugs. From the Bayesian perspective, initial models of drug sensitivity are learned assuming each feature has equal probability to be selected (uniform prior), and the subsequent sharing of models between drugs allows us to learn a feature selection prior for each drug. This iterative sharing between drugs is central to the learning power of CHER.

During each iteration we utilize L0-norm regularized regression to select predictive features for sensitivity to each drug. In the L0-norm regularized regression, a penalty is applied proportional to the number of features added to the model, as in classical stepwise regression methods, but the features added to the model are not shrunk as in lasso [25] or elastic-net [26]. L0-norm regularization has several advantages. First, the regularization term in the regression is nonparametric, since the sparse selection of predictors in L0-norm regularization is guided by the minimum description length (MDL), where selection of each feature is encoded as a *cost* or penalty that ensures sparsity of the model (Materials and Methods). Second, the correspondence between MDL and Bayesian statistics allows us to iteratively adjust our belief by setting the cost of each feature according to the probability of that feature being selected. At each iteration, we use L0-norm regularized regression with bootstrapping to build a probability distribution (prior) for each feature based on the number of times it was selected. This prior distribution is further adjusted by sharing information between drugs, constructing a penalty for feature selection in the next iteration (Fig 1B). Third, we use a greedy algorithm to efficiently construct an L0-norm normalized regression; the models resulting from this search have been demonstrated to have excellent performance [27]. The consideration of contextual predictors requires that the search space include the interaction between genomic features and contexts. While such large feature space may pose challenges for many algorithms, the greedy-search allows CHER to efficiently seek the relevant predictors in this large feature space.

**Fig 2:**
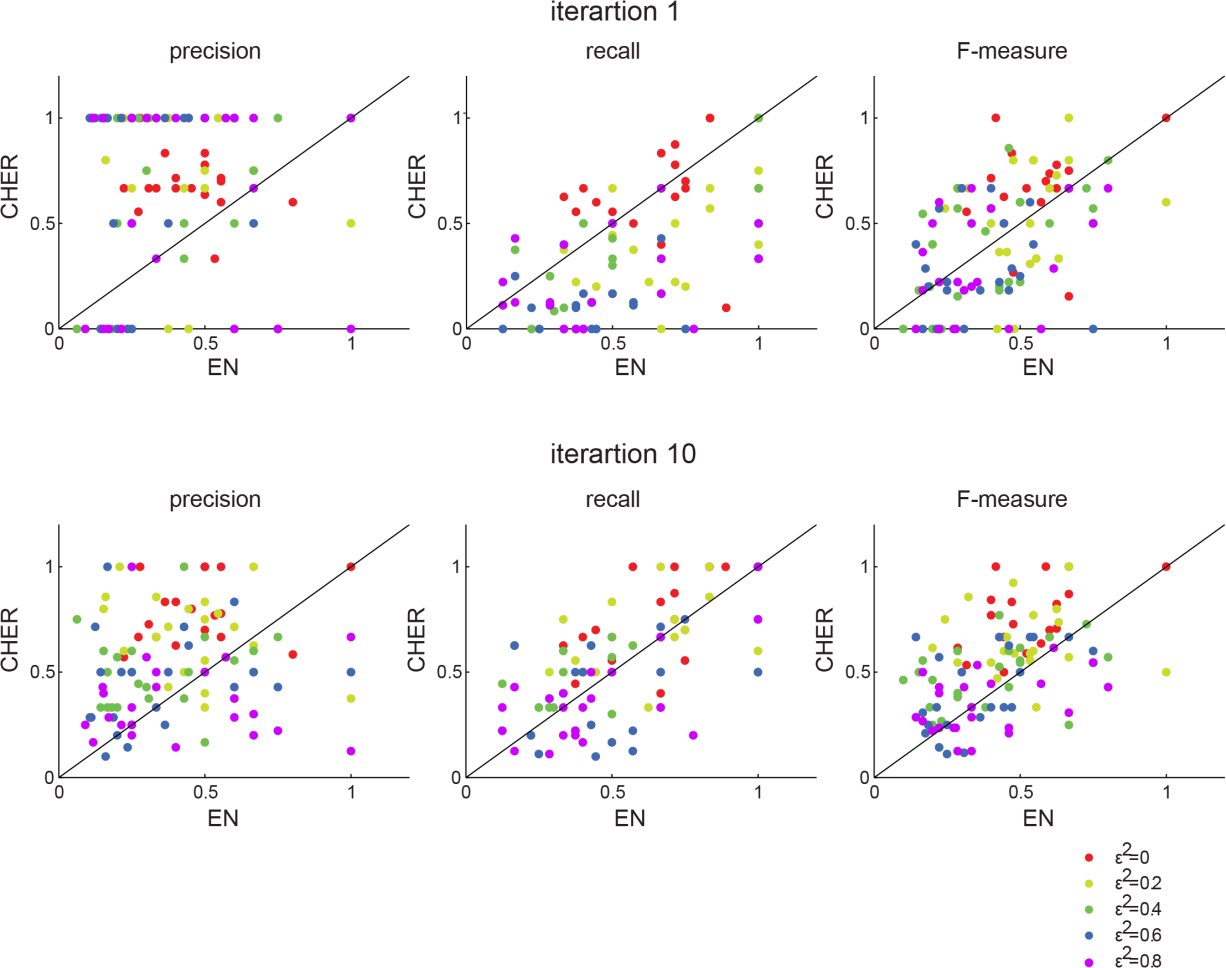
Comparison of performance of CHER and elastic net on synthetic data. Bootstrapped elastic net (EN) is compared to bootstrapped CHER. A threshold of 0.3 and 0.5 are applied to the relevant frequency (*τ*) to determine robust features in CHER and elastic, respectively. The precision, recall, F-measure of each phenotype from EN (x-axis) is plotted against that from CHER (y-axis). The first row shows the results of CHER from the first iteration and the second row the results of CHER from the 10^th^ iteration. Each dot represents a phenotype, colored by the noise level added.

To evaluate CHER’s performance, we test it on a synthetic dataset that is simulated from the real data (S1 Text). We compare CHER to the elastic net algorithm previously used for this data and evaluate three metrics: precision, recall, and F-measure (S1 Text, Fig 2). F-measure scores the harmonic mean of precision and recall and represents overall performance of the two algorithms. CHER trades off some recall to produce higher precision compared to the elastic net. In biological applications precision is often preferred to recall, since minimizing false positives saves future costly experimental validations. Thus, precision and F-measure scores in the final iterations suggest the overall superiority of CHER in identifying correct predictors (S1 Text and S4–S6 Figs).

### Application of CHER to CCLE datasets

CHER takes advantage of pooling samples from similar cancers to increase power. We constructed test datasets based on prior knowledge of cancer similarity and the number of available samples from each cancer type (S3 Fig), which largely constrained our selection. We pooled blood and lymphoid cancer cell lines (n = 70, CCLE-Blood) based on tissue origin. We pooled breast (n = 27) and ovary (n = 25) cancer samples (CCLE-BreastOvary) because of the genomic similarities between basal-like breast cancers and high-grade serous ovarian cancers [28]. Finally, among all available CCLE data, we further pooled together melanoma (n = 38) and glioma (n = 25) (CCLE-SkinGlioma) because melanocytes and neuroglia are both embryologically derived from ectoderm. Shared tumor-associated antigens [29] and dysregulated pathways [30] have been reported in melanoma and glioma. In addition, we observed high similarity between samples of central nervous system and skin tissues, as it is shown in the projection of samples on principal components derived from gene expression profiles (S7 Fig). Therefore, it is possible that these two cancers share some biological pathways or genomic features contributing to drug sensitivity.

Each dataset includes different numbers of possible split variables to specify potential contextual influences. In CCLE-SkinGlioma, only one possible split is allowed: whether a sample is glioma or not. In CCLE-BreastOvary, two possible splits are considered: we can separate samples by tissue origin (breast vs. ovary) or pathology (luminal breast cancer vs. basal-like breast and ovarian cancer). Finally, seven potential splits are considered in the CLLE-Blood subset, representing cancer types of different lineage origins (S1 Table). Two metrics are used to represent sensitivity to each drug: the concentration that inhibits 50% of proliferation (IC50) and the activity area above the curve fitted from the drug response data (ACT). The goals of CHER are to (1) identify the best split, if any, (2) select predictive genomic features that are common or context-specific (defined by the chosen split) for each drug sensitivity phenotype, and (3) learn the regression model to predict drug sensitivity.

Due to the small sample size, we further limit the possible features to decrease search space and therefore increase power. We compiled lists of genes associated with each cancer from literature and the Disease database [31]. Only mutation, copy number and gene expression of the genes associated with the analyzed cancers are included as potential predictors. S2 Table summarizes the number of phenotypes, features, and available samples in each dataset.

We evaluate CHER’s performance on the CCLE datasets with ten-fold cross-validation (Materials and Methods). Pearson and Spearman correlation coefficients are used to evaluate performance. The elastic net algorithm [26] is also applied to the CCLE subsets for comparison, as it has been successfully used to identify genomic features for drug sensitivity in [7, 8, 32]. Elastic net regression enables selection of predictive genomics features based on L1- and L2-norms; the latter is suitable for highly correlated gene expression features [32]. However, the naïve elastic net algorithm does not allow contextual predictors, and hence, each selected genomic feature is used to predict drug sensitivity of every sample, regardless of context. The application of elastic net here is the same as the setting in [7], where no contextual features were considered. To supplement the lack of contextual modeling in elastic net, the split variables used in CHER are also included as binary features in the feature pool for elastic net.

**Fig 3:**
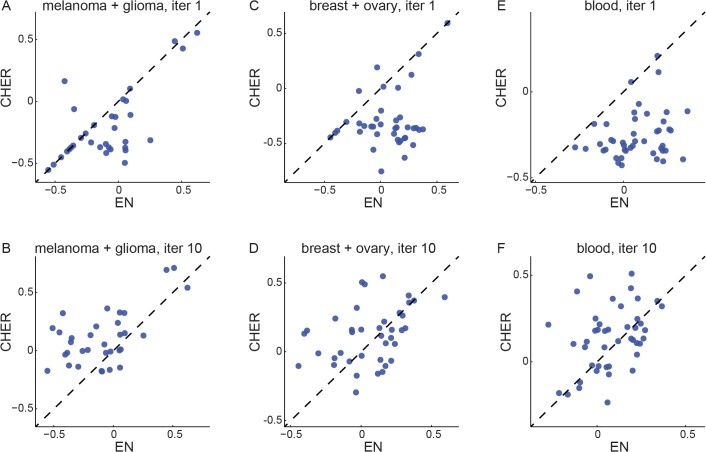
Comparison of performance between CHER and elastic-net (EN). Pearson’s correlation coefficients between the prediction and the true sensitivity data are calculated for each algorithm and plotted against each other (x-axis: elastic net, y-axis: CHER). Each dot represents a phenotype. A. Predictions for melanoma and glioma samples from the initial iteration of CHER algorithm are compared to those from elastic net. B. Predictions for melanoma and glioma samples from CHER after ten iterations are compared to those from elastic net. C., D. Similarly, but for breast and ovarian cancer samples. E., F. Similarly, but for blood samples.

Fig 3 compares the performance of CHER and the elastic net (Materials and Methods). As shown in Fig 3A, elastic net models outperform those from the first iteration of CHER. However, after ten iterations of sharing between models (Fig 3B), CHER shows significant improvement over elastic net. This is because the uniform prior used in the first iteration fails to yield models for many phenotypes. However, the performance is improved through additional iterations, as the information is exchanged between models of similar phenotypes and the priors of features are adjusted. The effect of transfer learning can already be observed in the second iteration (S8 and S9 Figs), demonstrating the utility of transfer learning between drugs of similar responses, as drugs that share similar targets often induce similar sensitivity (S10 Fig).

At the end of the iterative learning process, CHER gives better predictive performance (Pearson correlation coefficients, see Materials and Methods) than elastic-net for 60% (70/116) of the drug sensitivity phenotypes in all three datasets (p<6e-6, one tail paired t-test, for comparing Pearson correlation; p<2e-7 for comparing Spearman correlation; Fig 3, S8 and S9 Figs). Moreover, for these 70 phenotypes, the improvement of CHER’s prediction over elastic net is large, with a mean improvement of 0.24 in Pearson correlation (S11 Fig). Elastic net outperforms CHER in only 46 phenotypes with mean improvement of 0.12.

### Comparison of features selected by CHER and the elastic-net

To gain insight into the models CHER produces, we compare the features selected by CHER and elastic net. Both algorithms are applied to all samples in each dataset with bootstrapping. Only features that are robustly chosen via bootstrap are retained in the final model (Materials and Methods). Note there are many phenotypes for which the elastic net fails to select any feature because no features are chosen “frequently enough” among the bootstrap runs, indicating a lack of robustness in elastic net’s feature selection. For example, elastic-net fails to select any robust features for most phenotypes (35 out of 39) for CCLE-BreastOvary, whereas CHER only fails on one phenotype. Therefore, CCLE-BreastOvary is dropped from comparison. For the other two datasets, comparisons are made for a phenotype only when the elastic-net has also selected robust features following bootstrap.

**Fig 4:**
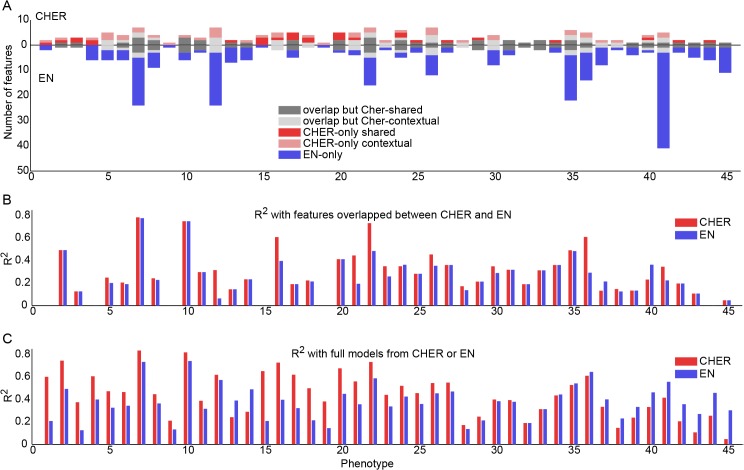
Comparison of features selected by CHER and elastic net (EN). A. Number of features selected by both and individual algorithms for each phenotype. For each phenotype (x-axis), numbers of features selected by CHER are represented on the positive y-axis whereas those selected by elastic net are represented on the negative y-axis. Features are separated into five groups, corresponding to features selected by both algorithms or by specific to individual algorithms. Phenotype 1–14 are from CCLE-SkinGlioma and the rest are from CCLE-Blood. B. Adjusted R^2^ of CHER and elastic net models using the features selected by both algorithms (features of the first two categories in A). C. As B, but all features selected by each algorithm are used. Phenotypes in all three figures are sorted by the difference of R^2^ between CHER and elastic net from C.

First, we compare the number of features chosen by each algorithm (Fig 4A). Compared to CHER, elastic-net often chooses many more features, likely due to the elastic net’s L2-norm regularization, which favors selecting correlated features. We compare the overlapping and unique features between the two algorithms by separating them into five categories: (1) features that are selected by both algorithms (*overlap but CHER-shared* in Fig 4A), (2) features that are selected by both but are only predictive for a subtype of samples in CHER (*overlap but CHER-contextual*), (3) features that are only selected by CHER and are predictive for all samples (*CHER-only shared*), (4) features that are only selected by CHER and are predictive only for a subtype of samples (*CHER-only contextual*) and (5) features that are only selected by the elastic net (*EN-only*).

From this decomposition, we find that 40/45 phenotypes have at least one feature that is selected by both CHER and elastic net. Using only these features, we estimate the variance explained (adjusted R^2^) by CHER and elastic net (Fig 4B). For CCLE-SkinGlioma (Phenotype 1–14 in Fig 4B), adjusted R^2^’s are similar between CHER and elastic net. This is because there are only two subtypes of samples in the data, and it can be encoded as a binary feature in the elastic net. However, when the subtypes of samples become more complicated as in CCLE-Blood, the merit of CHER’s models manifests in the gain of R^2^ (Phenotype 15–45 in Fig 4B). Even with the same set of selected features (categories 1 and 2 above), CHER explains more variance than elastic net for 12 phenotypes by considering contextual effects of the features.

When considering all features selected by each algorithm, we see CHER achieves better adjusted R^2^ than elastic net for 29/45 phenotypes (p<0.007, one-tail paired t-test, Fig 4C), even though CHER’s models often contain fewer features than the elastic net. CHER’s gains in R^2^ is also more significant than that of elastic net: CHER gains >0.2 R^2^ over elastic net for 11/29 phenotypes, whereas elastic net gains >0.2 R^2^ over CHER for 2/14 phenotypes. Together, the results suggest CHER’s final models explain more variance in the data, likely achieved through the modeling of context.

### Comparison with additional methods

In addition to the elastic net, we also compared the performance of CHER to the Multiple Inclusion Criterion (*MIC*) [27], multi-task lasso (*MTLASSO*) [33], the elastic net with all context-gene interaction features (*EN-INT*), and Bayesian multi-task multi-kernel regression (*BMKL*) that recently won the NCI-DREAM drug sensitivity prediction challenge [34]. MIC is an algorithm that selects features via the L0-norm and has demonstrated strong performance in feature selection and prediction tasks. It is the predecessor of CHER, as CHER extends MIC by adding transfer learning and context (Materials and Methods). MTLASSO is an extension of lasso that imposes the sparsity constraint on all learning tasks at once. It essentially shares features between all phenotypes. In contrast BMKL is a method that first uses multiple kernels for each data type (for example, mutation or gene expression) to summarize similarity between samples, and then uses Bayesian inference to learn regression weights on these to predict drug sensitivity [34]. An advantage of BMKL is that the regression models can be non-linear via kernel computations. Finally, we add all the cancer-type and gene interaction terms (contextual features) into the feature space and apply the elastic net with interactions (EN-INT). That is, we include in the feature pools the binary variables specifying cancer types and cancer-type specific features (eg. products of binary variables and genomic features) for EN-INT. Note all the split variables used in CHER are also included as binary features in the feature pool for all methods.

**Fig 5:**
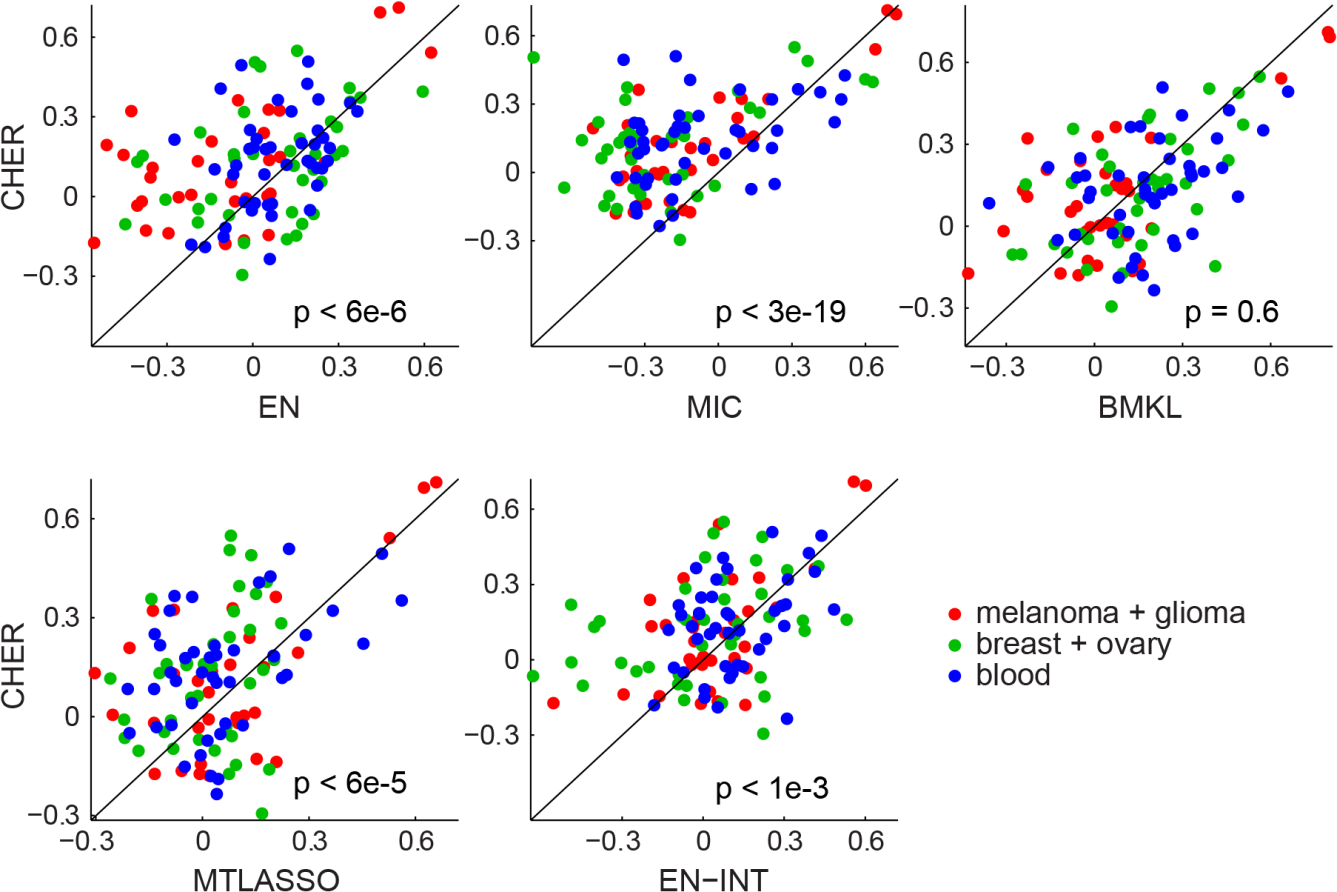
Comparison of CHER with other methods. Pearson correlation coefficients between the prediction and the sensitivity data are calculated for each algorithm. The correlation coefficients from each algorithm (x-axis) are compared to those from CHER (y-axis). Each dot represents prediction performance for one drug sensitivity. Method abbreviation: EN, the elastic net, MIC, multiple inclusion criterion; BMKL: Bayesian multi-task multi-kernel regression; MTLASSO: multi-task lasso; EN-INT: EN with context-gene interactions. P-values show the significance of CHER’s prediction compared to other methods (one-tail t-test).

We apply all methods to the CCLE datasets and compare their performance in a ten-fold cross-validation (Materials and Methods). Fig 5 and S12 Fig show the overall performance of each method. Across all three datasets, CHER outperforms most methods and performs comparably with BMKL. Specifically, CHER outperforms EN (p<6e-6, one tail paired t-test, for comparing Pearson correlation; p<2e-7 for comparing Spearman), MTLASSO (p<6e-5 for Pearson, p<2e-8 for Spearman), EN-INT (p<1e-3 for Pearson, p<3e-7 for Spearman) and MIC (p<3e-19 for Pearson, p<3e-24 for Spearman). CHER outperforms BMKL in CCLE-SkinGlioma (p<0.05 for Pearson, p<4e-3 for Spearman), has similar performance to BMKL in CCLE-BreastOvary, but BMKL performs better than CHER in CCLE-Blood.

These comparisons highlight the advantages of CHER. First, CHER outperforms EN-INT although all the contextual features are made available to the elastic net. This shows CHER’s superior feature selection, likely benefiting from transferring information between multiple phenotypes. Second, contextual features are important as CHER outperforms MIC even though CHER and MIC uses the same methodology for feature selection.

Despite the similar performance between CHER and BMKL, CHER also provides interpretability for the relationship between genomic features and drug sensitivity. In the three datasets, CHER identifies many predictive features that are either direct targets of the drugs or in similar pathways, suggesting the relationship between these features and drug sensitivity. For example, CHER identifies BRAF as a predictor for sensitivity to RAF inhibitor PLX4720 and MEK inhibitors (AZD6244 and PD-0325901) in CCLE-SkinGlioma; ERBB2 as a predictor for sensitivity to Lapatinib (EGFR and ERBB2 inhibitor) in CCLE-BreatOvary; ABL1 for sensitivity to ABL1 inhibitors (AZD0530, Nilotinib) in CCLE-Blood (S3–S5 Tables). This highlights CHER’s ability to derive models that not only are predictive of drug sensitivity but also helps elucidate mechanism of action.

### A Case Study of Sensitivity to Paclitaxel in Melanoma and Glioma Cell Lines

For the CCLE-SkinGlioma dataset, we pooled samples of melanoma (n = 38) and glioma (n = 25) together because melanocytes and neuroglia are both embryologically derived from ectoderm. PCA analysis also shows the similarity between the two types of samples (S7 Fig). Among the drugs screened, we found paclitaxel especially interesting because both melanoma and glioma cell lines show a wide range of sensitivity (ACT ranges from 3 to 7). Paclitaxel is a compound that targets tubulin and stabilizes the microtubule, leading to a defect in cell division. It has been used to treat various cancers, including melanoma, breast and ovarian cancers. In other cancers, sensitivity to paclitaxel has been associated with PI3K [35, 36], MAPK [37, 38] and NF-κB pathways [36, 39, 40].

**Fig 6:**
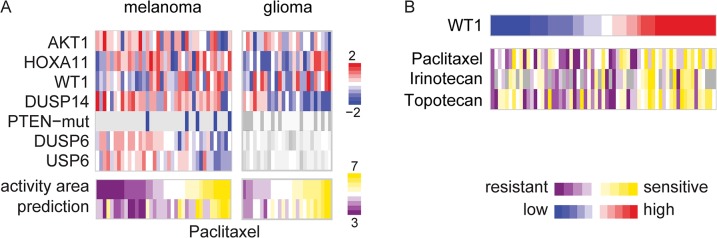
Example of predictive model for melanoma and glioma samples. A. CHER’s model for drug sensitivity to paclitaxel. Each vertical bar represents a data of a sample. All features are gene expression profiles except PTEN, which is a mutation profile feature (blue bars represents samples with mutations). AKT1 and WT1 are predictive for both melanoma and glioma. PTEN-mut, DUSP6 and USP6 are predictive features specific for melanoma whereas DUSP14 is specific for glioma. The greyed out heatmaps represents those features are not predictive for the samples. The predictions are obtained from leave-one-out procedure with the final selected features. B. Expression of WT1 is predictive of the cytotoxic drugs paclitaxel, irinotecan and topotecan, which likely due to IGF1-R activity.

CHER selects both shared and cancer-type specific features for the ACT phenotype of paclitaxel. Expression of both *AKT1* and *WT1* (Wilms tumor 1) are selected as shared predictors (Fig 6A). Interestingly, mutation of *PTEN* (phosphatase and tensin homolog), and expression of *DUSP6* and *USP6* (ubiquitin specific peptidase 6) are selected only for melanoma samples. CHER selection of *AKT1* and *PTEN* suggests the PI3K/AKT pathway is predictive of resistance to paclitaxel in melanoma cells.

In our model high expression of *DUSP6* and *DUSP14* is predictive of resistance to paclitaxel in melanoma and glioma. *DUSP6* and *DUSP14* suggest high levels of MAPK activity may be involved in paclitaxel resistance, since transcription of *DUSP6* (dual specificity phosphatase 6) is regulated by ERK [41, 42] and *DUSP14* modulates ERK activity and negatively regulates proliferation [43]. CHER’s prediction is supported by several studies that have shown that inhibition of ERK activity could increase sensitivity to paclitaxel in many cancers including colon, lung, and cervical cancers [35, 36]. Note that the elastic net fails to select any features for paclitaxel’s ACT phenotype.

### Resistance Predictor WT1 May Suggest Combined Therapy

Because several genes in our models seem to serve as proxies to pathway activity, we ask if the association between expression of genes and drug resistance may suggest combinatorial treatments. The assumption is, if a gene is predictive of hyper-activation of an oncogenic pathway and is associated with resistance to a drug, inhibition of the pathway may overcome the resistance and sensitize individuals to the original therapy.

In the CCLE-SkinGlioma dataset, CHER selects *WT1* expression as a predictor for paclitaxel and topotecan (S3 Table). Additional analysis shows that low expression of *WT1* is correlated with resistance to paclitaxel, topotecan, and irinotecan for all samples (Fig 6B). All three drugs are cytotoxic agents used in chemotherapy. *WT1* is known to negatively regulate transcription of *IGF-1R* (insulin-like growth factor I receptor) and activity of IGF-1R signaling can in turn activate MAPK and PI3K pathways [44]. Thus, we infer active IGF-1R signaling from low expression of *WT1*, which predicts resistance to each of the three chemotherapies. Since low expression of *WT1* implies high activation of IGF-1R, CHER’s model suggests that inhibition of IGF-1R may overcome the resistance to these chemotherapeutic agents. Indeed, beneficial effects of combining IGF-1R inhibitors with paclitaxel have been reported in non-small cell lung cancers [45–47], with topotecan in Wilms tumor cells [48], and with irinotecan in colon cancers [49, 50]. For glioma, inhibition of IGF-1R is shown to enhance the sensitivity to etoposide, which also targets topoisomerase [51].

## Discussion

Context plays an important role in biology. The interaction of context and genetics allows cells with the same genetic information to differentiate into diverse cell types geared towards various physiological functions. In cancer, contextual regulatory or signaling activities often influence cellular response to treatment. For example, subtypes of breast cancers show diverse sensitivity to drugs [52]. This presents an even greater challenge for understanding tumor biology and treatment when multiple subtypes are often found in the “same” cancer type. This may explain why a potent therapy often works for some patients, but fails in others. These challenges emphasize the need for context based precision medicine: treatments tailored for patients in the context of their genomic profile.

Current efforts to screen drug sensitivity on large collections of cancer cell lines [7, 8] provide a model system for precision medicine. However, since the number of samples for each cancer type is limited, computational analysis either has to neglect context or be underpowered by sample size. Additionally, despite the ongoing effort for drug screening with cell lines, the problem of sample size will remain as we discover more tumor heterogeneity and explore drug combinations. The naïve approach of learning predictive models for each drug independently will not have enough statistical power.

To tackle these problems, we proposed an algorithm that explicitly accounts for context and boosts its statistical power via transfer learning. The algorithm aims to identify both predictive features that are context-specific, as well as ones shared among all samples. Hence, we can pool samples of similar cancer types together, benefitting from the sample size to uncover shared features. Moreover, leveraging on the similarity between sensitivity profiles between drugs, CHER boosts learning power by transferring modeling information between “similar” drugs. This transfer of information allows CHER to learn which features are robustly predictive across multiple drugs, and helps reveal relations between drugs and pathways. For example, in CCLE-Blood data, the expression of *CRKL* is selected as a predictor to response to AZD0530 (target ABL), PF2341066 (c-MET), sorafenib (RTK), and nilotinib (ABL). CRKL is a substrate of BCR-ABL and plays an important role in leukemia [53, 54]. Without transfer learning between drugs, *CRKL* would not be chosen as a predictor for any of these drugs.

Our results demonstrate the importance of context and the value of explicitly modeling it into drug predictors. CHER gives better overall prediction performance compared to most methods tested and performs comparably with BMKL. While CHER and BMKL have similar predictive capability, CHER provides added value in its interpretive models. We show that dependence of drug sensitivity on MAPK pathway activity can manifest in expression of distinct genes in melanoma and glioma. In CCLE-Blood data where the relevant subtype for drug sensitivity is unknown, CHER learns the relevant classifications of samples to reveal the effects of context.

One of the CHER’s advantages is to select predictors that are common for similar cancer types. However, defining similar cancer types is not trivial and should be done with datasets where samples of cancer types are abundant. Here our selection of datasets is constrained by our knowledge of the cancer types as well as the number of available cell lines of each cancer type (S3 Fig). We believe with more data available, systematic analysis of similarity between cancer types can help us select which datasets to consolidate together for modeling.

Interestingly, we observe different degrees of similarity between cancer types in the datasets we used. For example, when we compare the pooled-cancer elastic net, single-cancer elastic net and CHER, we notice the difference of performances between the two datasets CCLE-SkinGlioma and CCLE-BreastOvary (S13 Fig). For CCLE-SkinGlioma, the single-cancer elastic net performs better than the pool-cancer elastic net, but the pool-cancer elastic net performs better than the single-cancer one in CCLE-BreastOvary. This may suggest the different degrees of ‘similarity’ between these cancers: breast and ovary cancers may share more similarity than melanoma and glioma. Nevertheless, CHER outperforms both elastic net models in both datasets (p<3e-3 and p<6e-7 for CHER vs. EN-single cancer and CHER vs. EN-pooled in CCLE-SkinGlioma, respectively; p<2e-3 and p<0.02 for CHER vs. EN-single cancer and CHER vs. EN-pooled in CCLE-BreastOvary, respectively; one tail paired t-test for comparing Spearman correlation).

Compared to other methods, CHER demonstrates outstanding performance and provides models that are straightforward to interpret. However, CHER currently has some limitations: Currently, we use 100 bootstrap runs and ten iterations of learning for robustness. However, we might be able to use fewer bootstrap runs and iterations to speed up the learning process (S14 Fig). Further research will be needed to determine how one can automatically determine the minimum number of bootstrap and iterative learning that are required. Additionally, although CHER searches among multiple potential splits for optimal splits, its current implementation only allows one split for each phenotype in the final model and only searches for predictors within a list of genes relevant to the disease. These restrictions serve to limit the model complexity and search space due to the paucity of current datasets. Nevertheless, these restrictions can be eliminated as data continues to accumulate. With the increase of sample size, CHER will have more power to search the entire genome for predictors and can be extended to consider multiple splits in each model.

## Materials and Methods

### Datasets

We take the data from Cancer Cell Line Encyclopedia (CCLE)[7] to study the association between drug sensitivity and both shared and context-specific genomic features among cancers. The cell lines were treated with one of 24 drugs at various concentrations. Sensitivity to each drug was characterized for each cell line. For our analysis, two phenotypes are considered for each compound: concentration that inhibits 50% of proliferation (IC50) and activity area above the curve fitted from the drug response data (ACT). Processed mutation and copy number data of the cell lines are obtained from the CCLE website (http://www.broadinstitute.org/ccle/home). Raw data of gene expression microarray are processed as follows. Samples are processed together with MAS5 algorithm [55]. Probes that are annotated to hybridize to multiple genes are discarded. Signals are then log transformed, and then we discard probes that have low (< 6) or high (> 15.5) values in more than 20% of the samples in a dataset. Principal component analysis is applied to z-scores for the remaining probes. Outlier samples are identified from the projection of samples on the first three principal components. Log-transformed data are then normalized according to the 75^th^ percentile within each sample. Finally, values of probes hybridized to the same gene are averaged to derive expression of the gene.

Three sets of data are extracted from the CCLE collection. The first set includes breast carcinoma (n = 27) and ovary carcinoma (n = 25) samples because of the genomic similarities between basal-like breast cancers and high-grade serous ovarian cancers [28]. The second set includes malignant melanoma (n = 38) and glioma (n = 25) samples, since melanocytes and neuroglia are both embryologically derived from ectoderm. Shared tumor-associated antigens [29] and dysregulated pathways [30] have been reported in melanoma and glioma. In addition, high similarity between samples of central nervous system and skin tissues is shown in the projection of samples on principal components derived from gene expression profiles (S7 Fig). Therefore, it is possible that these two cancers share some biological pathways or genomic features contributing to drug sensitivity. We refer to these two sets as CCLE-BreastOvary and CCLE-SkinGlioma.

The third set of samples extracted from CCLE includes cell lines derived from haematopoietic and lymphoid tissues (n = 70). This set includes samples of many different subtypes, including ALL (acute lymphoblastic leukemia), AML (acute myeloid leukemia), CML (chronic myeloid leukemia), plasma cell myeloma, and etc. Seven binary classifications are defined for samples and treated as potential relevant subtypes for the analysis (S1 Table). This set of data is designated as CCLE-Blood.

Since samples may be very resistant to the drug at the screened concentration in the CCLE data, many samples did not reach 50% inhibition of growth [7] and hence do not have valid IC50. Therefore, when more than 80% of samples do not have valid IC50, the IC50 phenotype of the drug is excluded from the analysis. When enough IC50 values are available, these are log transformed and treated as phenotype for modeling. Activity area (ACT) above the growth curve is treated as another phenotype associated with the drug sensitivity.

To limit the search space of predictors, we compile lists of genes associated with each cancer from literature and the Disease database [31]. Only mutation, copy number and gene expression of the genes associated with the analyzed cancers are included as potential predictors. Moreover, mutation data of genes with <10% mutated samples or wild-type are excluded. Genes with variance < 0.2 in their expression are filtered. Genes with variance < 0.15 in their copy number are filtered. S2 Table summarizes the number of phenotypes, features, and available samples in each dataset. S3–S5 Tables list all the phenotypes modeled by CHER in each dataset.

### CHER Algorithm

We developed an algorithm CHER (Contextual Heterogeneity Enabled Regression) that aims to uncover contextual predictive features for drug sensitivity. The algorithm gains statistical power by pooling samples of similar cancer types together for the analysis. Here we define a context as a cancer type, tissue type, or cancer subtype. We refer to this context as the *relevant subtype* that separates individuals into two groups where the predictive program of drug sensitivity can be different. Briefly, CHER is based on L0-norm regularized regression:

$$\underset{\beta }{\text{min}}\;{\left(y-X\beta \right)}^{2}+f\left({\left\|\beta \right\|}_{0}\right)$$ Eq 1

where *y* is the phenotype ($R^{n\times 1}$), *X* is the feature matrix ($R^{n\times p}$), *β* is a vector of coefficients ($R^{p\times 1}$) for the regression model and *f*(‖*β*‖_0_) is a function of L0-norm of *β*, serving as a penalty. The penalty function tends to yield a sparse solution where most elements of *β* are zeros. This allows us to select predictive genomic features for the phenotype. From an information theoretic perspective, the L0-norm penalty can be interpreted as the model description length. That is, when an entry in *β* is nonzero, we describe it with log_2_(p)+2 bits, where log_2_(p) bits are used to identify the index of the nonzero entry, and 2 bits are used to encode the actual coefficient [27]. Eq 1 then corresponds to finding a model of minimum description length (MDL) [56, 57], minimizing the fitting error while constraining the complexity of the model.

From Bayesian viewpoint, each feature has 1/*p* probability to be selected and have a nonzero coefficient [58]. This corresponds to a non-informative prior over the feature space while the minimization of sum of square errors correspond to the maximum of likelihood [59]:

$$\underset{\beta }{\text{max}}\;\text{log}\;P\left(y|X,\beta \right)+\text{log}\;P\left(\beta \right)$$ Eq 2

where *P*(*y*| *X*, *β*) ∼ *N*(*y*| *X β*, *σ* ^2^) with *σ* ^2^ as the variance of random noise and *P*(*β*) is the prior for the coefficients. The exchangeability between Eq 1 and Eq 2 represents the correspondence between the MDL (minimum description length) solution and the MAP (maximum *a posteriori*) estimate.

Extending Eq 1, CHER optimizes

$$\begin{array}{c} \underset{t,\beta^{S},\beta^{t1},\beta^{t0}}{\text{min}}\;\sum_{i}^{n}{\left(y_{i}-\sum_{j}^{p}x_{ij}\beta_{j}^{S}-\sum_{j}^{p}\delta \left(z_{it}=1\right)x_{ij}\beta_{j}^{t1}-\sum_{j}^{p}\delta \left(z_{it}=0\right)x_{ij}\beta_{j}^{t0}\right)}^{2}\\ +penalty\left(t\right)+f\left({\left\|\beta^{S}\right\|}_{0}\right)+f\left({\left\|\beta^{t1}\right\|}_{0}\right)+f\left({\left\|\beta^{t0}\right\|}_{0}\right) \end{array}$$ Eq 3

where *z*_*it*_ is a binary variable representing if sample *i* is of cancer type *t*, and *δ*(.) is an indicator function that returns 1 when sample *i* is of the cancer type queried. $\beta^{S}\left(R^{p\times 1}\right)$ are the coefficients for shared genomic features, whereas $\beta^{t1}\left(R^{p\times 1}\right)$ and $\beta^{t0}\left(R^{p\times 1}\right)$ are the coefficients for cancer-type-specific features. *n* and *p* represents the number of samples and the number of available genomic features, respectively. Fig 1A shows an example of model returned by CHER.

During CHER’s first iteration, we assume no prior knowledge or preference for feature selection. Therefore, we encode the penalty of each feature using log_2_(*p*). The penalty function *f*(.) multiplies this cost by the number of non-zero coefficients. For each subtype *t*, log_2_(*T*) is used as the *penalty*(t) where *T* is the total number of subtypes.

To take advantage of potential similarity between sensitivity to different drugs, CHER iteratively adjusts the penalty function *f* and *penalty(t)* according to its belief (prior probability) that a genomic feature or a split is relevant for the phenotype, by sharing information between predictive models. To enhance the robustness of feature selection, we use bootstrap. For each dataset (for example, CCLE-Blood), we align genomic features with phenotype so that each column of the two matrices presents a cell line. We then resample the cell lines (columns) with replacement to obtain a set of the original sample size (or the training set size during cross-validation). The bootstrap sampling procedure is done in advance so that the same subset of samples is used for training for all phenotypes. Then, during each iteration, CHER optimizes Eq 3 with the bootstrapped samples (100 bootstrapping runs). The frequency that each feature is selected for a phenotype is then used to estimate the prior probability of the feature being predictive:

$$P^{y}\;\left(\beta_{j}\neq 0\right)=\frac{\sum_{k=1}^{K}w_{yk}\tau_{jk}+a}{\sum_{k=1}^{K}w_{yk}+b}$$ Eq 4

where *k* enumerates phenotype, *τ*_*jk*_ is the frequency of feature *j* being selected for phenotype *k*, *a* and *b* are hyper-parameters of a non-informative beta prior (*a* = *b* = 0.5) to regulate this Bernoulli distribution, and *w*_*yk*_ is a similarity score between phenotypes *y* and *k*. We estimate *w*_*yk*_ by using sigmoid function to transform the Pearson’s correlation coefficient between phenotypes *y* and *k*. This prior probability is then transformed into penalty for the next iteration of learning. The prior probability of a subtype *t* being relevant is adjusted similarly (S1 Text). To ensure the final selected features are robustly predictive, CHER iterates the learning procedure ten times and the features are chosen according to the frequency *τ*_*jk*_ in the final iteration (*τ*_*jk*_ ≥ 0.3). Note for robust feature selection in elastic net, previous works [7, 8, 32] have suggested *τ*_*jk*_ ≥ 0.5. However, unlike elastic net, CHER is based on L0-norm, which does not select collinear features due to the lack of L2-norm regularization. To compensate for this, we use *τ*_*jk*_ ≥ 0.3. We demonstrated this threshold is appropriate to select robust features in our synthetic data analysis (S1 Text). We also chose the number of iteration (ten iteration) as the simulation results appear stable. Fig 1B shows CHER’s learning procedure. Details and pseudo-code of CHER algorithm are included in S1 Text. Source code of CHER is provided in S1 File.

### Comparison of Methods

We compare CHER to the elastic net [26], MIC [27], MTLASSO (multi-task lasso [33]) and to BMKL (Bayesian multi-task multi-kernel regression [34]), using ten-fold cross-validation to evaluate each algorithm’s prediction of drug sensitivity. During each “fold”, 9/10 of samples are used to learn predictive models for the phenotype, and predictions are made for the 1/10 of samples held out during training. The procedure is repeated ten times so that predictions for all samples are obtained.

For the elastic net, MTLASSO, MIC, bootstrapped samplings are used to train models as the procedure used for CHER. Because both the elastic net and MTLASSO require tuning of parameters, we optimize the parameters of these models through a nested cross-validation procedure: the parameters are selected within each “fold” using a nested ten-fold cross-validation optimizing mean square errors. To include only features that are frequently chosen in bootstrapping runs for elastic net, we apply different thresholds to *τ*_*jk*_ (frequency of feature *j* being selected for phenotype *k*). We use *τ*_*jk*_ ≥ 0.5 for final elastic net models since it retains robust features without being too conservative (no features would be chosen) or too lenient (S15 Fig). The same threshold was used MTLASSO. For MIC, because of its similarity to CHER, we use the same threshold *τ*_*jk*_ ≥ 0.3 to choose features that are frequently selected among bootstrapping runs.

In addition, we apply BMKL to the same datasets and features. Three kernels are used for mutation (Jaccard similarity coefficient kernel), copy number (Gaussian kernel) and gene expression (Gaussian kernel), respectively. Similar to the elastic net and MTLASSO, the parameters of BMKL are tuned through nested cross-validation procedure.

Pearson and Spearman correlation coefficients between the predictions and the phenotype data are calculated as evaluation metrics. When an algorithm fails to select any feature for a phenotype, the average of training data is used as prediction.

### Comparison of Features Selected by CHER and Elastic-net

To compare the features chosen by CHER and elastic net, we apply the algorithms to all samples in each data set. Bootstrapping is used to select robust predictors as described above. CCLE-BreastOvary is excluded from the comparison because elastic net fails to select any robust predictors. After the features are selected, we compare the overlaps between the two algorithms. In addition, adjusted R^2^ is used to evaluate the variance explained by the selected features.

## Supporting Information

S1 Fig Effects of tissue types on drug sensitivity. P-values are obtained with Kruskal-Wallis test for to assess the effects of tissue types on activity area (ACT) and log(IC50) of each drug. The dash line indicates the Bonferroni correction for p-value < 0.05. Tissues with fewer than 10 samples are excluded from the analysis.
(EPS)

S2 Fig Effect of context in drug sensitivity. A. Scatter plot of MDM2 expression (x-axis) and sensitivity (maximum activity area, AMAX; see Barretina *et al*. 2012) to Nutlin-3, each dot represents data of a cell line. Samples are colored based on the Pearson’s correlation coefficients between MDM2 expression and AMAX of Nutlin-3 are calculated for each tissue type. Tissues with correlation coefficients <-0.3 are colored red and the others blue. Correlation coefficients between MDM2 expression and Nutlin-3 for each group are shown in the figure. B. Scatter plots of MDM2 expression (x-axis) and sensitivity to Nutlin-3 (AMAX, y-axis) for samples of four tissue origins.
(EPS)

S3 Fig Number of cell lines of each tissue type in Cancer Cell Line Encyclopedia. Only cell lines with both available genomic profiles and drug sensitivity data are shown.
(EPS)

S4 Fig Accuracy of identifying the relevant subtypes (splits) in the synthetic data. The colored lines depict the accuracy of identifying the subtypes used to generate the data, as a function of the number of iterations. In order to decide if a chosen subtype is robust among bootstrapping runs, three different thresholds are used for the frequency (t) of a subtype being chosen among bootstrapping runs. Only the most frequently chosen subtypes that pass the threshold are called relevant. Each panel shows the results from data with different levels of noise.
(EPS)

S5 Fig Retrieval scores of relevant features for the synthetic data. Precision (the first row), recall (the second row) and F-measure (the last row) are used to evaluate the retrievals of features used to generate the synthetic data. The scores are plotted as a function of the number of iterations. Similar to S4 Fig, three different thresholds are used for the frequency (t) of a feature being regarded as relevant after the bootstrapping runs.
(EPS)

S6 Fig Effects of bootstrapping on precision. For each phenotype, the precision from the first iteration without bootstrapping (x-axis) is plotted against that from bootstrapped models (y-axis). Each dot represents a phenotype, colored according to the noise level added. A threshold of 0.3 is applied to the relevant frequency (*τ*) to determine significant features.
(EPS)

S7 Fig Similarity of gene expression profile between central nervous system and skin samples. Samples are projected to the first two principal components (PC1 and PC2) using principal component analysis with gene expression profiles. Samples of other tissues are omitted in the figure for clarity.
(EPS)

S8 Fig Comparison of performance between CHER and elastic-net (EN) using Pearson’s correlation coefficients. Pearson’s correlation coefficients between the prediction and the true sensitivity data are calculated for each algorithm and plotted against each other (x-axis: elastic net, y-axis: CHER). Each dot represents a phenotype. Results of CHER’s prediction from iteration 1, 2, 3 and 10 are shown here.
(EPS)

S9 Fig Comparison of performance between CHER and elastic-net (EN) using Spearman’s correlation coefficients. Spearman’s correlation coefficients between the prediction and the true sensitivity data are calculated for each algorithm and plotted against each other (x-axis: elastic net, y-axis: CHER). Each dot represents a phenotype. Results of CHER’s prediction from iteration 1, 2, 3 and 10 are shown here.
(EPS)

S10 Fig Similarity between phenotypes. Pearson’s correlation coefficients between the activity area (ACT) of each pair of drugs are displayed as heatmaps. The columns and rows are of the same order and are arranged by hierarchical clustering to group similar phenotypes together. Drug names and their targets (in the parentheses) are shown and indicate drugs with similar targets tend to group together.
(EPS)

S11 Fig Histograms of differences of performances between CHER and elastic net. A. Pearson’s correlation coefficients between the true data and the cross-validation predictions from CHER and elastic net are used to calculate the improvement of CHER’s prediction over elastic-net’s (CHER’s minus elastic-net’s). The histogram of the differences of Pearson’s correlation coefficients is shown. B. As A, but Spearman’s correlation coefficients between the true data and the predictions are used.
(EPS)

S12 Fig Comparison of CHER with other methods. Spearman correlation coefficients between the prediction and the true sensitivity data are calculated for each algorithm. The correlation coefficients from each algorithm (x-axis) are compared to those from CHER (y-axis). Each dot represents prediction performance for one drug sensitivity measurement. Method abbreviation: EN, the elastic net, MIC, multiple inclusion criterion; BMKL: Bayesian multi-task multi-kernel regression; MTLASSO: multi-task lasso; EN-INT: the elastic net with context-gene interactions. P-value from one-tail t-test is also reported.
(EPS)

S13 Fig Comparison of performance between CHER, the elastic-net using similar cancer samples (EN-pooled), and the elastic net using single cancer samples (EN-single cancer). The elastic nets are applied to CCLE-SkinGlioma and CCLE-BreastOvary. In EN-pooled models, samples of melanoma and glioma (or breast and ovary cancers) are modeled together. In EN-single cancer models, the elastic net is applied to each cancer type only. The correlation coefficients between the predicted values and the drug sensitivity from each model are calculated then sorted to compare the overall distribution.
(EPS)

S14 Fig Effects of bootstrap and iterative learning. A. The 75th-percentiles of Pearson’s correlation coefficients between the true data and the cross-validation predictions from CHER are shown as a function of the number of iteration; nbt: number of bootstrap sampling. B: Similar as A, but median data are shown. C, D: similar as A and B, but for Spearman’s correlation coefficients.
(EPS)

S15 Fig Comparison of performance between CHER and elastic-net (EN) using different thresholds to select robust features after bootstrapping. Different thresholds (0.3–0.8) are used for feature-selection frequency (*τ*_*jk*_ in Eq 4, main text) among bootstrapping runs for elastic net, in comparison with the threshold 0.3 used for CHER. For each algorithm with the specified threshold, the Pearson’s, Spearman’s correlation coefficients, and the number of robust features selected for each phenotype during the 10-fold cross-validation are sorted and plotted as curves. CHER constantly selects fewer features and achieves overall better prediction accuracy than elastic net. The threshold 0.5 is used for elastic net for final comparison in the main text.
(EPS)

S1 File CHER source code. (ZIP)

S1 Table Potential subtypes considered as split in CCLE-Blood. (XLSX)

S2 Table Summary of datasets. (XLSX)

S3 Table Features selected for CCLE-SkinGlioma. (XLSX)

S4 Table Features selected for CCLE-BreastOvary. (XLSX)

S5 Table Features selected for CCLE-Blood. (XLSX)

S1 Text Details of methods and algorithms are described. (PDF)
