# Supplementary material for: Context Sensitive Modeling of Cancer Drug Sensitivity
Source: PLoS One. 2015 Aug 14;10(8):e0133850. doi: 10.1371/journal.pone.0133850 (PMC4537214; doi:10.1371/journal.pone.0133850)
Supplement: S1 Text — (PDF) [file pone.0133850.s022.pdf]

S1 Text

Details on Estimation of Prior **2**

Search Algorithm **3**

Statistical Evaluation of CHER with Simulation **6**

Simulation *6*

Effects of Transfer Learning and bootstrapping *9*

Comparison with the elastic net *9*

Pseudo-codes for CHER **11**

Reference **15**

S1 Text

Details on Estimation of Prior

Similar as the penalty of features in regression (Eq. 4 in the main text), the penalty of the relevant subtype *t* for phenotype *y* corresponds to

$$P^y(t\ is\ the\ relevant\ subtype) = \frac{\sum_{k=1}^{K} w_{yk}\tau_{tk} + a}{\sum_{k=1}^{K} w_{yk} + b} \quad \textbf{Eq. S1}$$

where τ_{tk} represents the frequency of subtype *t* being chosen as the relevant subtype for phenotype *k*.

It is straightforward to estimate $P^y(\beta_j^S \neq 0)$ (Eq. 4 in the main text) using τ_{jk}^S (the relevant frequency of feature *j* for phenotype *k* across all samples). $P^y(\beta_j^{t1} \neq 0)$ and $P^y(\beta_j^{t0} \neq 0)$ can be derived similarly from Eq. 4 for subtype-specific features. Ideally, $P^y(\beta_j^{t1} \neq 0)$ and $P^y(\beta_j^{t0} \neq 0)$ should be estimated for each possible subtype *t*. However, when the number of possible subtypes *T* is large, it is impractical to estimate all the priors and infeasible to keep them in memory if the number of features *p* is large. Nevertheless, assuming the transference only occurs between similar phenotypes, we approximate $P^y(\beta_j^{t1} \neq 0)$ and $P^y(\beta_j^{t0} \neq 0)$ with Eq.4 using τ_{jk}^t (the relevant frequency of feature *j* for phenotype *k* for samples of subtype *t*) regardless of which subtype *t* is chosen as relevant in each run.

Search Algorithm

To optimize Eq. 3 in the main text, we adapted the greedy search algorithm proposed by (1). We provide the outlines of the algorithm here to illustrate our search strategy. Pseudo-codes are also provided in this document.

We use a forward-backward search strategy to select feature. That is, our search algorithm starts with forward search, adding one feature at a time if addition of the feature decreases the score defined by the objective function. Once the forward search finishes, we use backward search, removing one feature at a time if removal of the feature decrease the score. The search space includes all the genomic features and the context-gene interactions terms.

Forward search

During forward search, we evaluate the addition of each feature for each phenotype. Specifically, we evaluate the score of adding feature x to the current selected feature set X^c with

$$\mathrm{score}(X^c, x) = -\log P(y|\{X^c, x\}, \beta^c, \beta_x) + penalty(\{X^c, x\}) \qquad \textbf{Eq. S2}$$

where β^c and β_x correspond to the regression coefficient for X^c and x, respectively. The feature that results in the best (minimum) score is considered to be added to X^c if the resulting score is better than the previous score ($score(X^c)$). The penalty of each feature is –log2(p)+4 during the first iteration of CHER and -log2$(P^y(\beta_j \neq 0))$+4 after the first iteration where $P^y(\beta_j \neq 0)$ represents the prior estimated from the previous iteration. The constant 4 represents the coding cost to specify the regression coefficient (2 bits)(1)

and the cost to specify whether this feature is context-specific or not (2 bits). Note, if the best feature is the first context-specific feature to be added to the current model, this leads to the selection of the *split* variable (t in Eq. 3 in the main text) and limits the following search. Because Eq. S2 requires fitting of a regression model for each candidate feature at each iteration of the search, we facilitate the search by evaluating Eq. S2 only for the top 100 features that are mostly correlated with the residuals of the previous model.

One can stop the forward search if the best score from the current search is worse than the previous score. However, to avoid local minimum, we can also force the algorithm to continue adding additional features. Here we force CHER to search for five additional features even if adding the feature resulting in worse score. These features will be removed later during the backward search. Additionally, we stop the forward search if the variance of current residuals is too small (<1e-7).

Moreover, at the end of forward search, if the current model contains no context-specific features, we re-evaluate each feature in the current model to see whether any of them can be context-specific. Again, this is to avoid local minimum. We only make the features context-specific if it results in better score. If indeed any feature is made context-specific, we resume the forward search to see if we can add any other features to the model.

Backward search

During backward search, we evaluate removal of feature x from current feature set X^c by

$$\mathrm{score}(X^c \backslash x) = -\log P(y|\{X^c\}, \beta^c) + penalty(\{X^c\}). \qquad \textbf{Eq. S3}$$

If the score is better than score (X^c), we then remove x from current feature set. The backward search iterates until no removal of feature results in better score.

Equivalent-model testing

Sometimes the search algorithm may lead to a model that is more complicated than necessary. Specifically two cases may happen:

(1) Feature x is selected as a shared and a context-specific feature, but with the opposite signs of regression coefficients ($\beta_x^s \neq 0, \beta_x^{t0} \neq 0, \beta_x^s \cdot \beta_x^{t0} < 0$);

(2) Feature x is selected a context-specific feature for both subtypes, but with the same signs of regression coefficients ($\beta_x^{t1} \neq 0, \beta_x^{t0} \neq 0, \beta_x^{t1} \cdot \beta_x^{t0} > 0$).

Case (1) could indicate feature x have different effects (predictive powers) on two groups of samples. Another possibility is that feature x only has effects on group *t1* since β_x^{t0} can simply cancel out β_x^s for samples in group *t0*. For the latter, we can simply set both β_x^s and β_x^{t0} to zero and use β_x^{t1} to model the effect. This will reduce the penalty (one feature instead of two features).

Similarly, Case (2) may suggest feature x have different effects on two groups, but another possibility is that the feature in fact has the same effects on both groups ($\beta_x^{t0} = \beta_x^{t1}$). For the latter, we can remove both context-specific features and use β_x^s to model the effect.

Hence, we constantly check if such two cases occur during our greedy search. If detected, we explicitly test which case it should be so that we can obtain the simpler and hence less costly model.

Statistical Evaluation of CHER with Simulation

<u>*Simulation*</u>

We generated a synthetic dataset to test the proposed algorithm. In order to generate the data, the algorithm is first applied to CCLE blood cancer samples (n=70) where seven binary subtypes are defined. Mutation and gene expression features are used and 100 bootstrapping datasets are drawn to train the models for *activity area* of each drug. The whole training and bootstrapping procedure is repeated for ten iterations, and the priors are updated after each iteration. 0.15 is used as a frequency cutoff to select features after the final iteration. One phenotype did not have any features passing the threshold, so it was dropped from further analysis.

Of 23 phenotypes, 6 are set to have only shared features among all samples. That is, no subtype-specific features are used to simulate the sensitivity data for the 6 drugs. This is to test if the algorithm would only select shared features or if it would be biased to select subtype-specific features. The number of features used to synthesize phenotypes ranges

from 2 to 11, excluding intercepts. After the features are selected, the coefficients are estimated from the original data using ridge regression with $\lambda = 0.01$:

$$\min_{\beta} \ (y - X\beta)^2 + \lambda \|\beta\|_2^2 \qquad \textbf{Eq. S4}$$

The choice of ridge regression is to regularize the coefficients of collinear features. These coefficients are then used with the original features to simulate synthetic phenotype.

Gaussian-distributed noise is added to the simulated phenotypes. The noise is drawn from $N(0, \sigma^2)$, where $\sigma^2 = \varepsilon^2 \sigma_y^2$, proportional to the variance (σ_y^2) of synthetic data. ε^2 is set to 0.2, 0.4 ,0.6, and 0.8 to generate different levels of noise.

The algorithm is then applied to the simulated data. 100 bootstrapping datasets are drawn for modeling during each iteration, and 10 iterations are repeated to adjust the prior. The final model for each phenotype is determined by the relevant frequency of a feature (τ_{jk}). For the relevant subtype, only the one with the maximum relevant frequency passing a predefined threshold, θ, is considered significant and as the final relevant subtype. Three different threshold values, 0.3, 0.5 and 0.8, are used for θ to see the effects of the threshold.

In order to evaluate the algorithm, four metrics are calculated: split accuracy, precision recall and F-measure. Split accuracy is defined as:

$$split\ accuracy = \frac{\sum_{k=1}^{K} I(\widehat{t_k} = t_k)}{K} \qquad \textbf{Eq. S5}$$

where $\widehat{t_k}$ is the relevant subtype retrieved for phenotype k and t_k is the true relevant subtype used to synthesize the phenotype.

Precision and recall are defined for each synthetic phenotype *k*:

$$precision^k = \frac{\sum_{j=1}^{p} I(\tau_{jk} \geq \theta\ and\ \beta_j^k \neq 0)}{\sum_{j=1}^{p} I(\tau_{jk} \geq \theta)} \qquad \textbf{Eq. S6}$$

$$recall^k = \frac{\sum_{j=1}^{p} I\left(\tau_{jk} \geq \theta\ and\ \beta_j^k \neq 0\right)}{\sum_{j=1}^{p} I\left(\beta_j^k \neq 0\right)} \qquad \textbf{Eq. S7}$$

$$F\text{-}measure^k = \frac{2 \cdot precision^k \cdot recall^k}{precision^k + recall^k} \qquad \textbf{Eq. S8}$$

where θ is the threshold for relevant frequency, τ_{jk} is the relevant frequency of feature j, and β_j^k is the coefficient used to synthesize the phenotype. Here $\beta_{\cdot}^k$ includes both shared and subtype-specific features. Intuitively, precision measures how many of the selected features are truly relevant whereas recall indicates how many relevant features are recovered. There is usually a trade-off between precision and recall. For example, a lenient algorithm can select as many features as possible to achieve high recall, but its

precision would be low. In biology, high precision is often desired since it implies low false positive rate.

Effects of Transfer Learning and bootstrapping

To illustrate the effect of transfer learning, S4 and S5 Figs. shows the four metrics evaluating CHER's models as a function of iterations. For clear illustration, the scores are averaged across all phenotypes. We can see that all metrics improve after the first iteration. S6 Fig. shows the comparison of precision, recall and F-measure from CHER models with and without bootstrapping. The compared models are from the first iteration, controlling for the effect of transfer learning. As shown in the figure, bootstrapping significantly increases the precision of the algorithms regardless of the threshold for the relevant frequency.

Comparison with the elastic net

Barretina, Caponigro (2) and Garnett, Edelman (3) applied the elastic net regression to all samples. To evaluate the effect of considering context specificity, we applied the elastic net to the synthetic data. For the elastic net, 10-fold cross-validation is used to choose the parameters that optimize mean square errors (2). The Matlab implementation of elastic net, *lasso*, is used here (4). The range of 0.2 to 1 with increment of 0.1 is used for selecting the parameter *alpha*, which controls the ratio of L1 to L2 norm penalty

(*alpha=1* means no L2 norm penalty is used). The second parameter *lambda,* which controls sparsity, is chosen from the smallest to the largest possible values that give non-empty models. 100 bootstrapped runs of the elastic net are applied to each synthetic phenotype with the optimal parameters. All seven possible subtypes are treated as binary features and included for model training. For evaluation, we calculate precision and recall for the elastic net models. Since the elastic net does not select subtype-specific features, we simply evaluate the models regardless of subtype specificity.

Fig. 2 shows the comparison of the three metrics obtained from the CHER algorithm and the elastic net. CHER achieves higher precision than elastic net for most models at the first iteration (top panels). CHER's further improvement over the elastic nets can be seen in precision and F-measure after ten iterations of transfer learning (bottom panels). However, in exchange for high precision and hence low false positive rate, the CHER is more conservative than the elastic net. As a result, its recall is not as good as the elastic net. This is also due to the fact that the elastic net has the capability to select collinear features. Since the features used including gene expression, highly correlated features are expected. These results suggest the elastic net selects many more correlated features than the CHER algorithm to achieve high recall, and therefore suffers from low precision. Nevertheless, CHER outperforms the elastic nets in F-measure (harmonic mean of precision and recall), suggesting the overall better retrieval of the correct features.

The results from the simulation validate the correctness and robustness of the proposed algorithm. It also sheds lights on the effects of bootstrapping and transfer learning. These

results suggest the algorithm is relatively conservative due to the sparsity constraints. This is important since false positives can be costly in biology, especially when we want to pursue experiments to validate the roles of the selected features.

However, the CHER algorithm also suffers a similar issue as L1-norm regularized regression *lasso*. They cannot handle collinear features well. Correlated features would compete with each other during bootstrapping and decreases their relevant frequency to the phenotype. This argues for a lenient threshold for the relevant frequency. We will discuss this in the next section when the algorithm is applied to the real data.

Pseudo-codes for CHER

To help illustrate CHER algorithm, we provide the following detailed pseudo-codes. The syntax is mostly based on Matlab, but for brevity some non-Matlab syntax, such as "+=", may be used. CHER() is the function call for CHER algorithm modeling, whereas the rest of functions are called within CHER. For easy reading, function calls are heighted in bold and comments are highlighted in blue.

```
% CHER algorithm
function freq = CHER(A, X, Y, num_iter, weight)
    %input:
    % A: subtypes, binary matrix; X: features, Y: responses
    % A: n x T (matrix size), X: n x p, Y: n x m
    % weight: m x m, eg. similarity scores between Y's
    %return:
    % freq: frequency of feature selection at the
    %       final iteration
```

```
    % Step1: initial prior
    prior.subtype = repmat(1/T, T, m); % T x m matrix with value 1/T
    prior.shared_feature = repmat(1/p, p, m);
    prior.t1_feature = repmat(1/p, p, m); %as in Eq.3
    prior.t0_feature = repmat(1/p, p, m); %as in Eq.3

    % Step 2: obtain bootstrap samples before modeling
    for bi = 1:num_bootstrap
        %subsample with replacement
        bootstrap_sample(:,bi) = resample(1:n, n, true);
    end

    % iterative learning
    for iter = 1:num_iter
        %initialize frequency of feature selection
        freq.subtype = zeros(T,m);
        freq.shared_feature = zeros(T,m);
        freq.t1_feature = zeros(T,m);
        freq.t0_feature = zeros(T,m);

        % Step 3: for each y, use bootstrap samples to obtain the
        % frequency of feature selection
        for yi = 1:m
            for bi = 1:num_bootstrap
                sample_index = bootstrap_sample(:,bi);
                A_bt = A(sample_index,:);
                X_bt = X(sample_index,:);
                y_bt = Y(sample_index,yi);
                [beta,subtype] = CHER_regress(A_bt, X_bt, y_bt, prior);

                % update the frequency of feature selection
                freq.subtype(subtype, yi) += 1/m;
                freq.shared_feature(beta(1:p) ~= 0,yi) += 1/m;
                freq.t1_feature(beta(p+1:2*p) ~= 0,yi) += 1/m;
                freq.t0_feature(beta(2*p+1:3*p)~=0,yi) += 1/m;
            end
        end

        % Step 4: update prior based on the frequency of feature
        % selection
        prior.subtype = CHER_prior(weight,freq.subtype,0.5,0.5);
        prior.shared_feature = …
            CHER_prior(weight,freq.shared_feature,0.5,0.5);
        prior.t1_feature = CHER_prior(weight,freq.t1_feature,0.5,0.5);
        prior.t0_feature = CHER_prior(weight,freq.t0_feature,0.5,0.5);

    end
end


% CHER_regress: solve regression model Eq. 3
function [beta, selected_subtype] = CHER_regress(A, X, y, prior)
    %input:
    % A: n x T, X: n x p, y: n x 1
    % first column of X contains all one (intercept)
    %return:
    % beta: regression coefficients)
```

```
    % selected_subtype: the relevant subtype

    % data initialization
    model.A = A;
    model.X = [X, get_interaction_term(A,X)]; % n x (p+ T*p*2)
    model.y = y;
    model.selected_subtype = 0; % no relevant subtype

    % initialize regression coefficients: 1~p: linear features;
    % p+1~2*p and 2*p+1~3p are subtype t1 and t0 features as in Eq.3
    model.beta = zeros(p*3, 1);

    % model initialization: yhat = mean of y
    model.beta(1) = mean(y);
    cur_score = negloglike(model) + penalty(model, prior);

    % Step 1: forward greedy search: add one feature that improves the
    % score most at each iteration
    for xi = 1 : p*3
        new_model = forwardsearch(model, prior);

        %negloglike: negative log likelihood of the regression
        new_score = negloglike(new_model) + penalty(new_model, prior);

        if new_score < cur_score
            model = new_model;
            cur_score = new_score;
        else
            %stop forward search
            break;
        end
    end

    % Step 2: backward greedy search: try to remove one feature in the
    % modelthat has least contribution to the scores
    while true
        new_model = backwardsearch(model, prior);
        new_score = negloglike(new_model) + penalty(new_model, prior);
        if new_score < cur_score
            model = new_model;
            cur_score = new_score;
        else
            break %stop backward
        end
    end
    beta = model.beta;
    selected_subtype = model.selected_subtype;
end


% CHER_prior: calculate prior, Eq. 4
function prior = CHER_prior(weight, selected_frequency, a, b)
    %input:
    % a, b as in Eq.4
    % weight: m x m (number of tasks), w in Eq.4
    % selected_frequency: 3*p x m, tau in Eq.4
    %return:
```

```
    % prior: 3*p x m

    % matrix multiplication
    prior = selected_frequency * weight;

    % calculate as Eq. 4, prior(j,i): prior of feature j
    % selected for task k
    prior = prior + a;
    for k = 1:m
        prior(:,k) = prior(:,k) / (sum(weight(:,k)) + b);
    end
end

% penalty: calculate penalty (Eq. 3) from priors
function cost = penalty(model, prior)
    cost = 0;
    for i = 1:3*p
        % if a feature is selected (non-zero coefficient), add its
        % penalty
        if model.beta(i) ~= 0
            cost = cost + (-log2(prior(i)));
        end
    end
end


% stepwise feature selection: forward search
function new_model = forwardsearch(model, prior)
    % search the feature increases the score most

    % no split is selected yet
    if model.selected_subtype == 0
        % search best feature among all (p + T*p*2) features
        best_feature = search_best_feature_and_subtype(model, prior);
        if best_feature is subtype-specific
            model.selected_subtype = subtype(best_feature);
        end
    else
        % split was already selected
        % search best feature among p + 2*p
        %(limited by the already selected subtype)
        best_feature = search_best_feature_fix_subtype(model, prior);
    end
    new_model = add_feature(model, best_feature);
end

% stepwise feature selection: backward search
function new_model = backwardsearch(model, prior)
    % remove the feature that results a minimal score change
    score = Inf;

    for i = 1:3*p

        % if feature i was selected, try to remove feature i and
        % recalculate the score to see if it's better
        if model.beta(i) ~= 0
```

```
            model_remove_one = remove_feature(model, i);
        end

        new_score = negloglike(model_remove_one) +
            penalty(model_remove_one, prior);

        % removal of feature i gives better score
        if new_score < score
            new_model = model_remove_one;
        end
    end
end
```

Reference

1. Dhillon PS, Foster D, Ungar LH. Minimum description length penalization for group and multi-task sparse learning. The Journal of Machine Learning Research. 2011;12:525-64.
2. Barretina J, Caponigro G, Stransky N, Venkatesan K, Margolin AA, Kim S, et al. The Cancer Cell Line Encyclopedia enables predictive modelling of anticancer drug sensitivity. Nature. 2012;483(7391):603-7. Epub 2012/03/31. doi: 10.1038/nature11003. PubMed PMID: 22460905; PubMed Central PMCID: PMC3320027.
3. Garnett MJ, Edelman EJ, Heidorn SJ, Greenman CD, Dastur A, Lau KW, et al. Systematic identification of genomic markers of drug sensitivity in cancer cells. Nature. 2012;483(7391):570-5. Epub 2012/03/31. doi: 10.1038/nature11005. PubMed PMID: 22460902; PubMed Central PMCID: PMC3349233.
4. The MathWorks. MATLAB and Statistics Toolbox Release 2012b.
